# Kinetic changes in virology, specific antibody response and imaging during the clinical course of COVID-19: a descriptive study

**DOI:** 10.1186/s12879-020-05549-8

**Published:** 2020-11-10

**Authors:** Qiu-jing Wang, Yan-zhen Yao, Jun-shuai Song, Qiao Wang, Li-yun Xu, Zhou-jun Bao, Dai-wen Mao, Ji-hang Zhou, Zhe-en Zhang, Yan Wang, Yi-wei Li, He-ping Wang, Lue Li, Hai-yan Pan, Guo-qiang Zhang, Shi-bo Li

**Affiliations:** 1 Department of Infectious Diseases, Zhoushan Hospital, Wenzhou Medical University, NO.739 Dingshen Road, Zhoushan City, 316021 Zhejiang China; 2 Department of Clinical Laboratory, Zhoushan Hospital, WenZhou Medical University, Zhoushan, 316021 Zhejiang China; 3 Department of Emergency, Rushan People’s Hospital, Shandong, 264500 Rushan China; 4 Key Laboratory of Medical Molecular Virology (MOE/NHC/CAMS), School of Basic Medical Sciences, Shanghai Medical College, Fudan University, Shanghai, 200032 China; 5 Cell and Molecular Biology Laboratory, Zhoushan Hospital, Wenzhou medical University, Zhoushan, 316021 Zhejiang China; 6 Department of Radiology Diagnostic Center, Zhoushan Hospital, WenZhou Medical University, Zhoushan, 316021 Zhejiang China; 7 Department of Respiratory Medicine, Zhoushan Hospital, WenZhou Medical University, Zhoushan, 316021 Zhejiang China; 8 Department of Gastroenterology, Zhoushan Hospital, WenZhou Medical University, Zhoushan, 316021 Zhejiang China; 9 Department of Gastrointestinal Surgery, Zhoushan Hospital, WenZhou Medical University, Zhoushan, 316021 Zhejiang China

**Keywords:** SARS-CoV-2, COVID-19, Antibody response and qRT-PCR, Lung image

## Abstract

**Background:**

To explore the kinetic changes in virology, specific antibody response and imaging during the clinical course of COVID-19.

**Methods:**

This observational study enrolled 20 patients with COVID-19, who were hospitalized between January 20–April 6, 2020, in the two COVID-19 designated hospitals of Zhoushan, Zhejiang and Rushan, Shandong, China, The laboratory findings, imaging, serum response to viral infection, and viral RNA level in the throat and stool samples were assessed from onset to recovery phase in patients with COVID-19.

**Results:**

SARS-COV-2 RNA was positive as early as day four. It remained positive until day 55 post-onset in the sputum-throat swabs and became negative in most cases (55%) within 14 days after onset. Lymphocytopenia occurred in 40% (8/20) of patients during the peak infection period and returned to normal at week five. The most severe inflammation in the lungs appeared in week 2 or 3 after onset, and this was completely absorbed between week 6 and 8 in 85.7% of patients. All patients had detectable antibodies to the receptor binding domain (RBD), and 95% of these patients had IgG to viral N proteins. The antibody titer peaked at week four. Anti-S IgM was positive in 7 of 20 patients after week three.

**Conclusions:**

All COVID-19 patients in this study were self-limiting and recovered well though it may take as long as 6–8 weeks. Our findings on the kinetic changes in imaging, serum response to viral infection and viral RNA level may help understand pathogenesis and define clinical course of COVID-19.

**Supplementary Information:**

The online version contains supplementary material available at 10.1186/s12879-020-05549-8.

## Key points

1. This study systematically and comprehensively describes the dynamic changes of nucleic acid, imaging and serum antibodies in COVID-19.
2. The viral RNA level was the highest at the early stage of onset.
3. 5%patients do not produce IgG to viral N proteins.

## Background

Coronavirus disease 2019 (COV-19) is an emergent infectious disease caused by severe acute respiratory syndrome coronavirus − 2(SARS-COV-2) [1]. Since the outbreak of COVID-19 in December 2019 in Wuhan, China, this has quickly spread in the six continents of Asia, Europe, North America, South America, Oceania and Africa, and became a global pandemic [2–4].

SARS-COV-2 is the seventh member of the enveloped coronavirus family under the genus beta-coronavirus [1, 3]. SARS-COV-2 shares 96 and 79.5% homology with bat coronavirus and severe acute respiratory syndrome coronavirus (SARS-COV), respectively [2, 3]. SARS-COV-2 encodes four structural proteins: spike (S), membrane (M), envelope (E), and nucleocapsid (N) proteins. The S protein in the homotrimer is required for binding cellular receptors [5], and both the M and E protein are involved in virus assembly [6]. The main function of the N protein is to encapsidate viral genome to form capsids, and act as a viral RNA silencing suppressor to facilitate viral replication [7]. Furthermore, the N protein is highly immunogenic, and is overexpressed during infection [8]. SARS-COV-2 is an emergent pathogen, and is highly transmissible from person to person. COVID-19 elicits the antibody response of both IgM and IgG forms in infected individuals. The emergence of two antibodies is an indicator of disease improvement. The detection of the IgM antibody is of diagnostic value for SASR-COV-2 infection, and a switch from IgM to IgG usually takes 11–14 days [9]. However, merely limited knowledge is available on kinetic changes in humoral immune response over the disease course in patients with COV-19, especially for the SARS-COV-2 neutralizing antibody profile.

The present study analyzed the kinetic changes in the clinical manifestation, virologic and serologic response to SARS-COV-2, and the chest imaging from onset to recovery, in a study of patients with COVID-19. In addition, the neutralizing antibodies against the receptor binding domain of angiotensin I converting enzyme 2 (ACE2), which is a cellular receptor for SARS-COV-2 entry, were analyzed.

## Methods

### Patients and study outline

In this study we followed the guidelines on the strengthening the Reporting of observational studies in epidemiology (STROBE). Twenty patients with COVID-19, who were hospitalized between January 20 to March 2, 2020, in two hospitals designated for providing COVID-19 care in Zhoushan hospital (15 patients), Zhoushan, Zhejiang and Rushan People’s Hospital (5 patients), Rushan, Shandong, China, were enrolled into this descriptive study. All patients were followed up to April 6, 2020. SARS-COV-2 infection in all patients was confirmed by SARS-COV-2 RNA positivity, performed by the local CDC test center. The COVID-19 diagnosis and clinical classification follows the COVID-19 diagnosis criteria and treatment plan (seventh edition) issued by the National Health Commission, China [10], and the consensus for diagnosis and prevention of COVID-19 in children [11].

Clinical specimens including Sputum throat swab, blood, and stool were collected periodically (2–10 days interval) after admission. The study outline is presented in Supplementary Figure 1.

Epidemiological data which include patients demographic characteristics, coexisting disease, history of close contact were obtained with standardized investigation forms, clinical and radiological characteristics, laboratory findings, daily clinical manifestations, clinical course, patient vital signs and prognosis data were obtained from the medical records of each patient. All data were verified by a physician and two clinical assistants.

The present study was approved by the Ethics Committee of Zhoushan Hospital (document no.2020–003, 2020–004), and a written informed consent was obtained from each of 20 patients or their parents.

### Real-time reverse transcription polymerase chain reaction (qRT-PCR)

The total RNA in the swab samples was extracted using a QIANGEN nucleic acid extraction kit. Then, the RNA was mixed with 4 ul of qRT-PCR enzyme mixture and 4 ul of primer probe 2019-nCOV (ORF1ab/N) for cDNA synthesis, with reverse transcription at 50 °C for 10 min. Then, this was subjected to 40 cycles of PCR amplification at 96 °C for 10 s, and at 55 °C for 40 s. The positive cycle threshold (Ct) value was set at < 37, the negative Ct value was set at > 40, and the indeterminate Ct value was set at 37–40.

### Antibody assay

A fully automatic chemiluminescence analyzer (Shenzhen New Industry Biomedical Engineering Co., Ltd.) that contains a modular biochemical immunoassay system that supports conducting a fully automated chemiluminescence immunoassay was used. SARS-COV-2 recombinant antigen (capture antigen) was used to coat the magnetic microspheres, and these were distributed into wells. Pre-diluted serum or control samples were added, followed by the addition of the ABE * labeled anti-human IgG monoclonal antibody (detection antibody). Then, the substrate solution was dispersed to the wells for the chemiluminescence reaction to generate an optical signal. Afterwards, the relative light intensity (RLU) in each well was read. The RLU signal was generally proportional to the specific antibody concentration in the serum. This was classified as reactive when the optical signal was ≥1.10 AU/ml, non-reactive when the optical signal was < 0.900 AU/ml, and gray area when the optical signal was within 0.900–1.100 AU/ml.

### Enzyme-linked immunosorbent assay (ELISA) of the neutralizing antibodies to RBD

Fifty microliters of coating buffer containing S1 and N proteins [1] at 10 μg/ml were dispersed to each well, and incubated at 4 °C overnight. On the following day, 200 ul of blocking buffer was added, and this was incubated at room temperature (RT) for two hours. Then, 50 ul of each of the tested serum samples were added and incubated at RT for one hour. The conjugated secondary antibody (1:5000; Thermo Fisher Scientific, 31,410) at a volume of 50 ul was dispersed to each well, and incubated at RT for one hour. The detection was visualized after the addition of 50 ul of ABTS (Thermo Fisher Scientific, 00–2024), and the optical density was read. The antibody concentration in serum was calculated according to the formula derived from the standard curve.

### Data analysis

Illness onset was defined as the first day of the reported symptoms consistent with COVID-19, while the first positive viral RNA was defined as the first day of onset of asymptomatic. Categorical variables were presented as numbers and percentages, and continuous variables were presented as the mean and standard deviation, when these were normally distributed. The patient’s laboratory indicators, nucleic acid dynamic changes, and IgG and IgM dynamic changes were represented by a line chart.

## Results

### Epidemiologic and clinical characteristics of patients with COVID-19 (Table 1)

A total of 20 patients with COVID-19 were enrolled into the present study. Among these patients, 12 patients were male and eight patients were female, and their age ranged within 7–67 years old. This participants of 20 patients consisted of three asymptomatic, two mild, 13 moderate and two severe infections. Among the 13 moderate patients, three patients had underlying diseases, which included one patient with hypertension, one patient with diabetes, and one patient with hypertension and rheumatoid arthritis. Six patients had a direct travel history to Wuhan, four patients had close contact with COVID-19 patients, and 10 patients had family clustering. The main clinical manifestations included cough in 16 patients, fever in 11 patients and fatigue in 10 patients. Two severe patients experienced reduced oxygen consumption. Diarrhea presented as the first symptom, which remained for 12 days in one of all the study participants. Three patients were given high-flow humidified oxygen inhalation, and seven patients were given nasal catheter oxygen inhalation. No mortality occurred in this study.

**Table 1:** Characteristics of the 20 patients with COVID in Zhoushan (the values represent the numbers [percentages], unless stated otherwise)

Characteristics	All patients (***n*** = 20)	Mild (***n*** = 2)	Moderate (***n*** = 13)	Severe (***n*** = 2)	Asymptomatic (***n*** = 3)
**Average age, years**	39.3 ± 15.3	31 ± 4	43.3 ± 11.5	65.5 ± 0.5	9.7 ± 1.8
**Age group (years)**					
≤ 18	4 (20)	0 (0)	1 (7.7)	0 (0)	3 (100)
19–59	11 (55)	2 (100)	9 (69)	0 (0)	0 (0)
≥ 60	5 (25)	0 (0)	3 (23)	2 (100)	0 (0)
**Gender**					
Male	12 (60)	1 (50)	7 (54)	2 (100)	2 (67)
Female	8 (40)	1 (50)	6 (46)	0 (0)	1 (33)
**Epidemiology history**					
Direct contact with Wuhan	6 (30)	0 (0)	5 (38.5)	1 (50)	0 (0)
Close contact	4 (20)	1 (50)	3 (23.0)	0 (0)	0 (0)
Family gathering	10 (50)	1 (50)	5 (38.5)	1 (50)	3 (100)
**Coexisting disease**					
Diabetes	1 (5)	0 (0)	1 (7.7)	0 (0)	0 (0)
Hypertension	2 (10)	0 (0)	2 (15.4)	0 (0)	0 (0)
Rheumatoid arthritis	1 (5)	0 (0)	1 (7.7)	0 (0)	0 (0)
**Symptoms**					
Cough	16 (80)	1 (50)	9 (69)	2 (100)	0 (0)
Fever	11 (55)	1 (50)	8 (62)	2 (100)	0 (0)
Fatigue	10 (50)	2 (100)	6 (46)	2 (100)	0 (0)
Others	2 (0)	0 (0)	0 (0)	2^ab^	0 (0)
**Oxygen way**					
Nasal cannula	7 (35)	0 (0)	7 (87.5)	0 (0)	0 (0)
High flow oxygen	3 (15)	0 (0)	1 (12.5)	2 (100)	0 (0)
**Prognosis**^c^					
Complete recovery	16 (80)	2 (100)	11 (85)	0 (0)	3 (100)
Incomplete recovery	4 (20)	0 (0)	2 (15)	2 (100)	0 (0)

^a^:two patients with chest tightness; ^b^: one patient with diarrhea; ^c^: up to April 6

### Clinical courses of patients with COVID-19

**Fig. 1:**
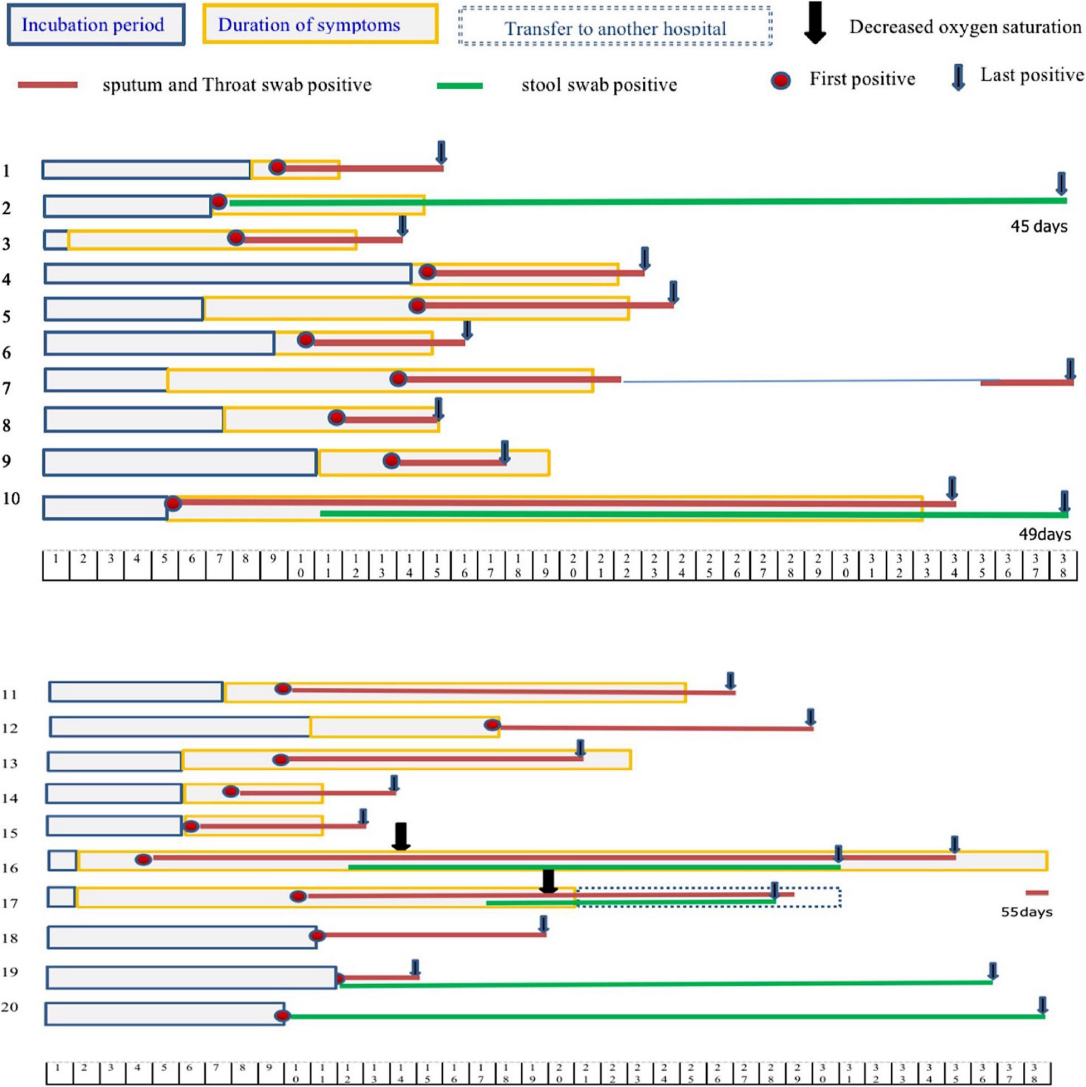
Individual clinical course of the 20 cases with COVID-19

In the present study, the incubation period ranged within 1–14 days, with an average of 6.8 ± 2.8 days. The symptoms improved within one week for mild patients, 5–27 days (average: 11.5 ± 5.3 days) for moderate patients, and 27–37 days (average: 32 ± 5 days) for severe cases. The viral nucleic acid positivity in the pharyngeal and sputum swab varied within 4–53 days, and 11 patients became negative within two weeks. A total of six patients had persistent nucleic acid positivity in the stool samples, which lasted for 27–49 days after onset (Fig. 1).

**Fig. 2:**
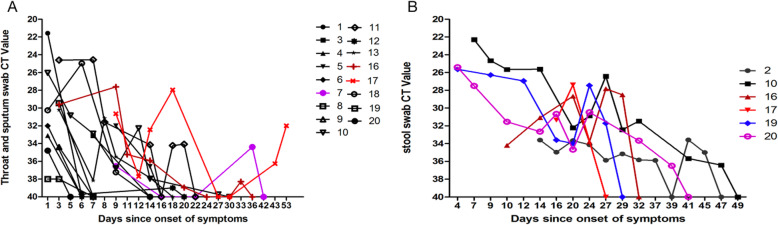
Kinetics changes in the viral RNA level of SARS-COV-2 in the sputum and throat swabs (**a**) and stool swab (**b**) in the present study of 20 patients. Note for Fig. A: No data was included for patient 2, 14 and 15 due to the viral RNA negativity in the sputum and pharyngeal swabs, or the absent Ct value. Patient 16 and 17 (red) were diagnosed with severe COVID-19. The viral RNA was negative, and was subsequently positive in patient 7 (purple). Patient 19 and 20 were asymptomatic

Viral RNA was detected in the pharyngeal and sputum samples of 17 patients. The Ct value for the viral RNA level was the smallest at the early stage of onset, suggesting a high viral load. The Ct value gradually increased, as reflected by the reduced viral load over time. The viral RNA became negative in the pharyngeal swab in seven days (seven cases, including three asymptomatic, one mild, and three moderate cases), 14 days (two cases), and 15–30 days (five moderate cases). Interestingly, three patients who were negative for viral RNA for the initial two consecutive times subsequently became positive. Patient 7 was negative for viral RNA until week two, and remained positive until day 42. Patient 16 became positive after week one, and remained positive until day 36. Patient 17 became positive at day 43, and remained positive until day 53. Patient 11, 12 and 13 were negative for viral RNA in the stool samples. Among the six patients with viral RNA positivity in the stool samples, patient 2 had detectable viral RNA in the stool until day 47. Patient 20 was positive for viral RNA in sputum from day 4 to day 41, and remained positive in the stool samples until day 49 (Fig. 2).

### Kinetics changes in the laboratory and radiographic findings in patients with COVID-19

**Fig. 3:**
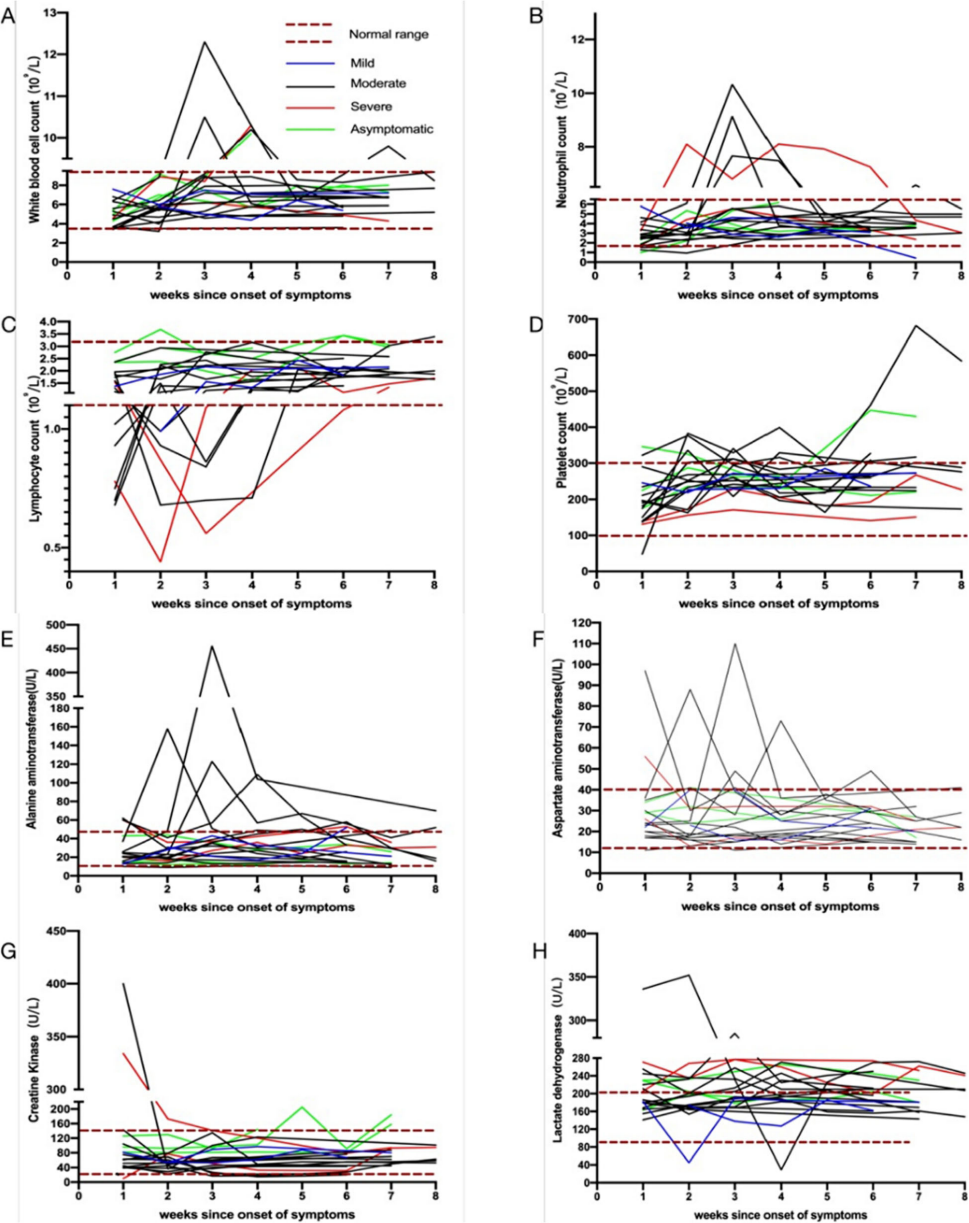
Kinetics of the laboratory findings for patients with COVID-19

The kinetic changes in leukocyte, neutrophil, lymphocyte and platelet count, alanine aminotransferase, aspartate aminotransferase, creatine kinase and lactate dehydrogenase were assessed during the 8-week period (from onset to week eight) in 20 patients. White blood cells (WBC) and neutrophils were within the normal range or slightly decreased upon onset. An increase in WBC and neutrophils at week three was observed in patient 10 and 13. The WBC and neutrophils of all patients returned to normal levels at the fifth week (Fig. 3a and b). A decrease in lymphocyte count was detected in eight patients (40%). Patient 16 and 17 had the lowest lymphocytes at week 2 and 3 (Fig. 3c). The platelet count was significantly elevated in patient 10, while this remained normal in the remaining patients (Fig. 3d). Patient 5, 7, 8 and 10 exhibited an increase in alanine aminotransferase (ALT) and aspartate aminotransferase (AST) during the course of the disease, accounting for 20% (4/20), while the remaining patients were approximately within the normal range (Fig. 3e and f). Creatine phosphokinase (CK) mainly presented with abnormalities on patient 5, 17 and 18. However, this was within the normal range for all patients, except for patient 18, at week three (Fig. 3g). There was an increase in LDH within four weeks of onset in five patients (25%). Among these patients, patient 16 and 17 returned to normal at week seven (Fig. 3h).

### Computed tomography (CT) imaging

**Fig. 4:**
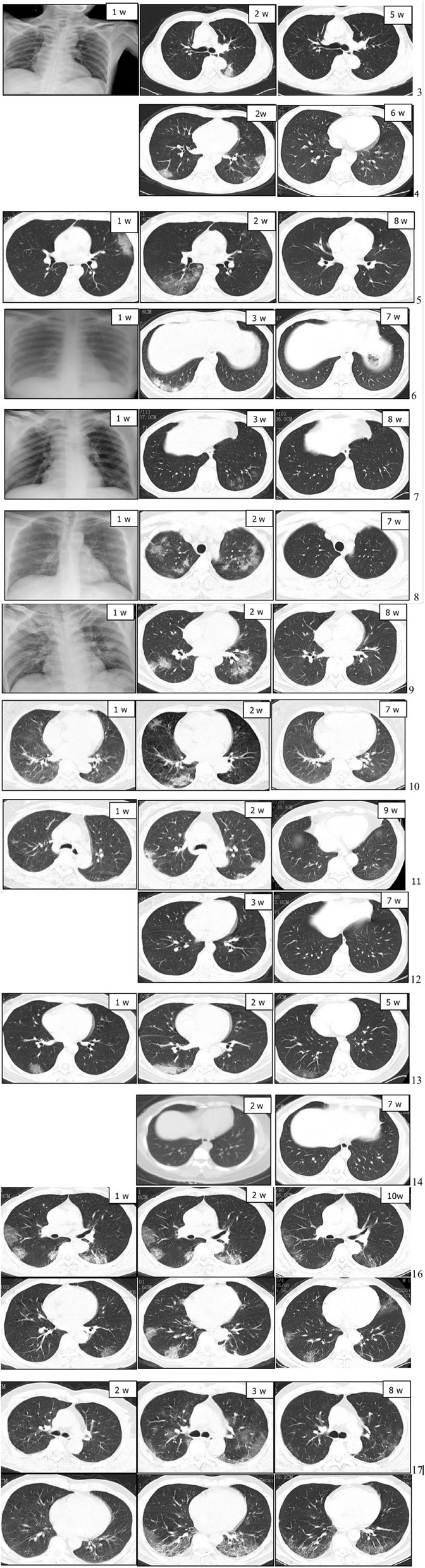
Kinetic changes in the chest CT imaging findings for patients with COVID-19.Case 3 at the initial stage of COVID-19 (week 1 after onset) shows the inflammation in the middle and lower lobes of the right lung, and the increase in flake density in the middle lobe of the right lung and lower lobe of the left lung at week 2. The inflammation in both lungs was completely absorbed at week 5. Case 4 at the initial stage of the disease (week 1) shows the unremarkable CT imaging, and the local inflammation detected in the lower left lobe of both lungs at week 2, which was absorbed at week 6. Case 5 at the initial stage of the disease (week 1) shows the infection in the left upper lobe and right lower lobe, which remained in these locations with an expanded range at week 2. This decreased to scattered inflammation (presented as a thin shadow) in the upper lobe of the left lung, and was absorbed in the right lung at week 8. Case 6 at the initial stage of the disease (week 1) shows the normal CT imaging, and the pneumonia in the lower lobe of the right lung at week 3. There was no inflammation in both lungs at week 7. Case 7 at the initial stage of the disease (week 1) shows a tiny inflammation in the lower left lung. The pneumonia scattered in both lungs at week 3, and was completed absorbed at week 8. Case 8 at the initial stage of the disease (week 1) shows a tiny inflammation of the upper left lung. This was scattered with flakes, some lesions were actually transformed in both lungs at week 2, and these were absorbed with the formation of local fibrosis at week 7. Case 9 at the initial stage of the disease (week 1) shows the increased and thickened texture in both lungs, the patchy shadows in the lower part of two lungs, and the shadowed glass density. The inflammatory lesions disappeared, but localized fibrosis occurred at week 8.Case 10 at the initial stage of the disease (week 1) shows the tiny flake inflammation in both lungs, and the dense and consolidated scattered flake ground glass in both lungs at week 2. Most of the inflammation absorbed with residual lesion and local fibrosis at week 7.Case 11 at the initial stage of the disease (week 1) shows the dense scattered shadow of flaky ground glass in both lungs, and the dense scattered flake ground glass that expanded in both lungs at week 2. This was almost completely absorbed at week 6. Case 12 (no lung CT at week 1) presents a scattered flocculent density with increased shadow and blurred edges, which could be mainly observed in the left lower lobe at week 3. This was absorbed in both lungs at week 7.Case 13 at the initial stage of the disease (week 1) shows the dense flaky ground glass in the lower lobe of both lungs. The ground glass lesion expanded in the range, but the density decreased in the lower lobe of both lungs at week 2. The inflammation was absorbed in the left lung, and residual inflammation occurred in the right lung at week 5.Case 14 (no week 1 CT) presents a tiny inflammation in the lower part of the right lung at week 2, which was completely absorbed at week 7. Case 16 at the initial stage of the disease (week 1) shows a scattered membrane glass shadow, which was mainly in the outer zone of both lungs, and a new lesion was detected in addition to the original lesions at week 2. There were high-density patchy and striated shadows on both lungs with fibrosis at the convalescent stage. Case 17 at the initial stage of the disease (week 1) shows the ground glass lesion outside the lower lobe of both lungs. The extent of the lesion increased, and this was scattered in the flake density with increased shadow. Some of the lesions were consolidated, especially under the pleura in both lungs, at week 2, with scattered flake density and increased shadow. Some of the consolidated lesions become narrower with the fibrosis in both lungs at the convalescent stage

All patients underwent chest X-rays or chest CT scans. Five of these patients had no pneumonia changes, and one patient came from a field without CT imaging data. The chest CT imaging was available in 14 of 20 patients (the CT imaging was not available for patient 1, 2, 15, 18, 19 and 20). Among the 14 patients with CT imaging, 12 patients were abnormal and two patients were severely abnormal. A single lung lobe was infected in two (14.3%) patients, two lung lobes were affected in five (35.7%) patients, three lung lobes were affected in three patients (21.4%), four lung lobes were affected in two patients (14.3%), and five lung lobes were affected in two patients (14.3%) (Table 2). Lung inflammation was noted in the CT imaging of 10 patients at week 1 after onset. The pneumonia of 10 patients reached a peak at week 2, while the pneumonia of four patients reached a peak at week 3. The CT imaging of 10 patients returned to normal by week 7 or 8, while residual pneumonia was observed in patient 10 and 11. Notably, many patchy and cord-like high-density hyperplasias, with localized fibrosis trends, remained in both lungs in two severe cases (patient 16 and 17), and these patients continued to cough (Fig. 4).

**Table 2:** The lung lobes affected in the 14 moderate and severe COVID-19 cases (*n* [%])

Lobes involved	Moderate (*n* = 12)	Severe (*n* = 2)	*N* (%)
Single lobe	2 [6, 14]	0	2 (14.3)
Two-lobe	5 [3, 4, 7, 12, 13]	0	5 (35.7)
Three-lobe	3 [5, 10, 11]	0	3 (21.4)
Four-lobe	1 [9]	1 (50%) [16]	2 (14.3)
Five-lobe	1 [8]	1 (50%) [17]	2 (14.3)

[]: Case number

### Kinetic changes in specific antibodies in patients with COVID-19

**Fig. 5:**
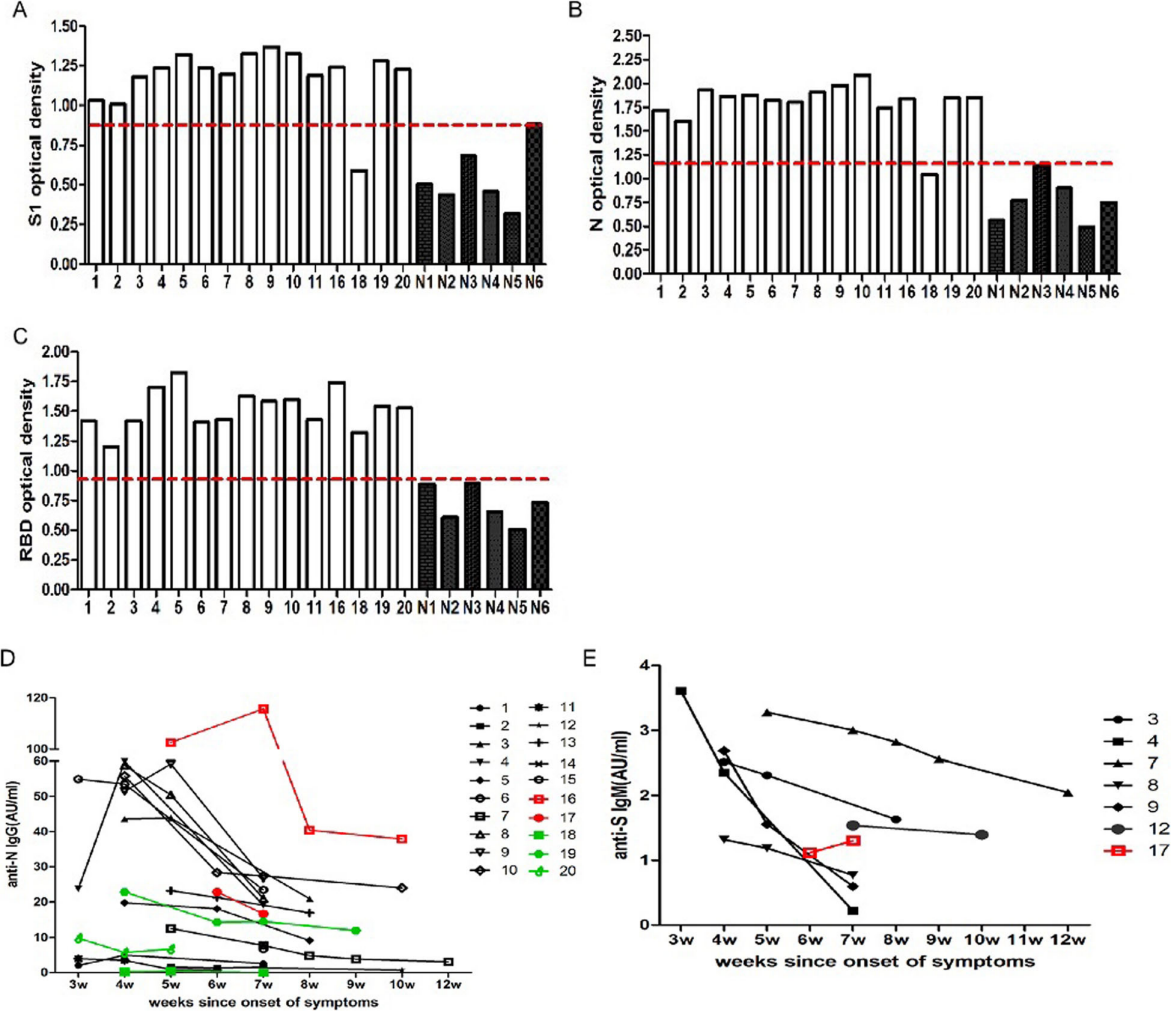
Kinetic changes in serum antibody titers from week 3 to week 12. Patients were numbered from 1 to 20, while healthy controls numbered from N1 to N6. S1-IgG (**a**), N-IgG (**b**) and RBD-IgG (**c**) shows the OD value at week 3 or 4, while **d** and **e** shows the kinetic changes at the anti-N IgG and anti-S IgM antibody level

Both serum specific IgG antibodies to the S1 and N protein were detected in 14 of 15 patients (93%) at week 3 or 4 after onset. Patient 18 was negative (Fig. 5a and b). All 15 patients were positive at a high titer for the IgG antibody to the RBD domain of the S protein (Fig. 5c).

The serum anti-S IgM and anti-N IgG in 20 patients were assessed after week 3 (recovery phase) (Fig. 5d and e). Since week 3, merely 7 of 20 cases had a detectable anti-S IgM antibody. The IgM level gradually decreased over time, and became negative in three patients at week 7, and the IgM positive rate was merely 20% at week 7. However, the IgM level was stable in patient 7, and remained positive at week 12 (Fig. 5e). IgG was detected in 19 of 20 patients, and merely patient 18 was negative. The serum IgG level peaked at week 4, and declined over time in 19 patients. Two mild cases had low IgG levels. The low IgG level detected in patient 11 became negative at week 5, and patient 12 became negative at week 10. A significantly high anti-N IgG was detected in patient 16, who was severely ill (Fig. 5d).

## Discussion

In the present study, 4 of 20 COVID-19 patients could be traced back to Wuhan, where COVID-19 initially spread, while the remaining patients likely acquired the disease within the family, or through close contact [12, 13]. The average incubation period for COVID-19 infection in the present study was 6.8 days. Main clinical manifestations included cough (94%), fever (73.%) and fatigue (52.9%). One exception was patient 17, who started with diarrhea, and did not have any respiratory symptoms throughout the course of disease.

All three cases (15%, 3/20) with asymptomatic infection were children, and these cases comparable to the reported 15.8% asymptomatic infections among children [14]. These cases were positive for viral RNA in the sputum-throat swabs, which only lasted for a short duration, despite being positive until day 32 in stool samples obtained from two of them. The long duration of viral RNA positivity in stool has raised the question of “fecal spread”, although this has yet to be confirmed [15]. Furthermore, these cases had normal profiles in the hematologic and biochemical tests, and there was no pneumonia in the lung imaging.

Our date suggested that the duration of SARS-COV-2 virus RNA positivity in stool swab was longer than that in respiratory specimens, However, whether the positive nucleic acid in the stool is infectious remains to be established. The viral RNA positivity in fecal sample reappeared in one case and remained positive until day 43, probably reflecting a false negativity or sample error in the early detection.

The clinical course of two severe patients lasted for 32 days, and average respiratory detoxification time was 39 days. The clinical course of one of these patients was 55 days, while this was longer than the reported 37 days in both cases [16]. This patient fully recovered, suggesting that long detoxification may not signal poor outcomes. The viral RNA level in these two patients was the highest at week 1 after onset, suggesting the peak viral load at week 1, and a decline over time [17–19]. However, the conditions of these patients worsened during the following two weeks, suggesting that the severity of COVID-19 was not correlated to the decrease in viral load in these two cases [16].

All patients had low numbers of white blood cells upon onset. Eight patients (8/15) experienced lymphopenia, and two severe patients had the lowest lymphocyte count. However, they returned to normal at week 4. This was consistent with the previously reported correlation between lymphocyte count and disease prognosis [20, 21].

Abnormal liver function was detected in some patients, suggesting the possible liver damage by COVID-19, although the drug factor could not be completely ruled out [22, 23]. The liver function returned to normal at week 7 or 8. Notably, two severe patients presented with both liver and myocardial injury [20]. One patient was transferred to the intensive care unit (ICU) at day 17, but returned to normal within 4 weeks.

The lung imaging revealed that most of moderate patients had lungs with < 3 affected lobes, and severe and a few moderate patients had lungs with > 4 affected lobes, This may indicate that the extent of lung infection largely determined the severity of the disease. These lung lesions most frequently appeared between week 2 and 3, which was consistent with that of 6–11 days reported by Wang et al. [24], and the pneumonia was completely absorbed between week 7 and 8, except one severe and one moderate patient in whom extensive lung inflammation lingered.

The major structural protein of SARS-COV-2 consists of spike glycoprotein (S, Spike Protein), small envelope glycoprotein (E, Envelope Protein), membrane glycoprotein (M, Membrane Protein) and nucleocapsid protein. The RBD domain in the S protein has been shown to bind ACE2, which is a cellular receptor for SARS-COV-2 entry [25]. The antibody to RBD was considered to neutralize the SARS-COV-2 infection. In the present study, it was found that all 15 infected patients had detectable anti-RBD neutralizing antibody at week 3 or 4. However, the serum antibody level in each patient varied, reflecting the different capacities to produce this neutralizing antibody. IgM was not detected in the three cases with asymptomatic infections after week 3, and anti-N IgG in 1 (patient 18) of 3 patients remained negative after week 3, but did had a detectable anti-RBD antibody, indicating that not all antibodies can be elicited in infected individuals [26]. Two mild patients were negative for IgM, but were positive for IgG at a relatively lower titer.

As noted, the SARS-COV-2 IgM antibodies was positive in 4 (24%) of 17 patients at week 4, and remained positive in three cases (15%) at week 7, suggesting the short lifespan for the IgM antibody. This was in line with the reports of previous studies [9], suggesting an average of 12 days of IgM positivity. IgM could be detected after one month in a few cases.

Previous studies have shown that the specific IgG antibody can be detected in 14 days [9]. In the present study, the specific IgG was detected in 91.7% (11/12) of patients at week 4, and this also peaked at week 4. One patient became IgG negative at week 5, and another patient became negative at week 10. However, the remaining patients had a detectable IgG during the study course of 12 weeks. In addition, the serum IgG antibody level declined over time. Antibody detection can supplement the qRT-PCR-based diagnosis of COVID-19.

SARS-COV-2 RNA in the pharyngeal and sputum can stay positive for 42 days, suggesting the infectiousness when transmitted. A sufficient quarantine period is required to ensure that no new transmission occurs among the recovered COVID-19 patients.

Most of the COVID-19 patients are hospitalized at week 2 or 3 after onset in China. The Chinese Health Department requires patients who are willing to donate convalescent plasma to be discharged after week 2, ensuring the protective neutralizing antibody at the peak. As reported, the SARS antibody titer may peak for four months after the onset, and decrease within six months [27]. However, the high IgG titer declined rapidly in the present study, and this was no longer delectable in two patients at week 5 and 10, respectively, supporting the protocol for the early collection of blood from convalescent patients who are likely have a high level of specific neutralizing antibody [26]. This also raises a critical question of whether the immunity after COVID-19 is long enough to manage long-term infections. The present data suggest that some of these patients may have a risk for reinfection with SARS-COV-2 due to the decline in the specific IgG protection antibody [28].

## Conclusions

In summary, the present study of COVID-19 in the Zhoushan area found revealed that COVID-19 consisted of asymptotic, mild, moderate and severe forms, and approximately 10% of the cases were severe. All 20 cases recovered well without specific antiviral treatment. The severer disease, the longer the time required for recovery, implying the longer infectiousness for transmission. Since the stool contained viral RNA longer than the throat swabs, a longer disinfection of stool is required. The pneumonia in most of the COVID-19 patients can be resolved within 7–8 weeks. Most patients developed humoral immunity to SARS-COV-2. IgM was detectable in only a small fraction of these patients at week 4, and the IgG peaked at week 4 and remained positive at least until week 12. The decline in antibody level in blood over time suggests the waned protection, which may make these recovered patients vulnerable to SARS-COV-2 reinfection.

Nevertheless, this study contains several limitations. First, We only enrolled in relatively small number of sample. Second, the question whether the virus in the stool was contagious remained inconclusive. Third, some severe patients revealed certain degree of pulmonary fibrosis during the recovery period. An extended follow up is needed to determine if the observed pulmonary fibrosis would affect respiratory function. Fourth, the duration and protection efficiency of RBD antibodies post recovery also require additional investigations in future studies.

## Supplementary Information

**Additional file 1: Supplementary Figure 1.** The study design and data collection flow chart.

## Data Availability

The datasets generated and analyzed during the present study are available from the corresponding author on reasonable request.

## Abbreviations

ACE2: Angiotensin I converting enzyme 2
ALT: Alanine aminotransferase
AST: Aspartate aminotransferase
CK: Creatine phosphokinase
COV-19: Coronavirus disease 2019
E: Envelope
M: Membrane
N: Nucleocapsid
RBD: Receptor binding domain
RLU: Relative light intensity
RT: Room temperature
SARS-COV-2: Severe acute respiratory syndrome coronavirus − 2
SARS-COV: Severe acute respiratory syndrome coronavirus
S: Spike
WBC: White blood cells
