# Supplementary figures and images for: Kinetic changes in virology, specific antibody response and imaging during the clinical course of COVID-19: a descriptive study
Source: BMC Infect Dis. 2020 Nov 10;20:818. doi: 10.1186/s12879-020-05549-8 (PMC7652587; doi:10.1186/s12879-020-05549-8)

**Supplementary Figure 1**  The study design and data collection flow chart


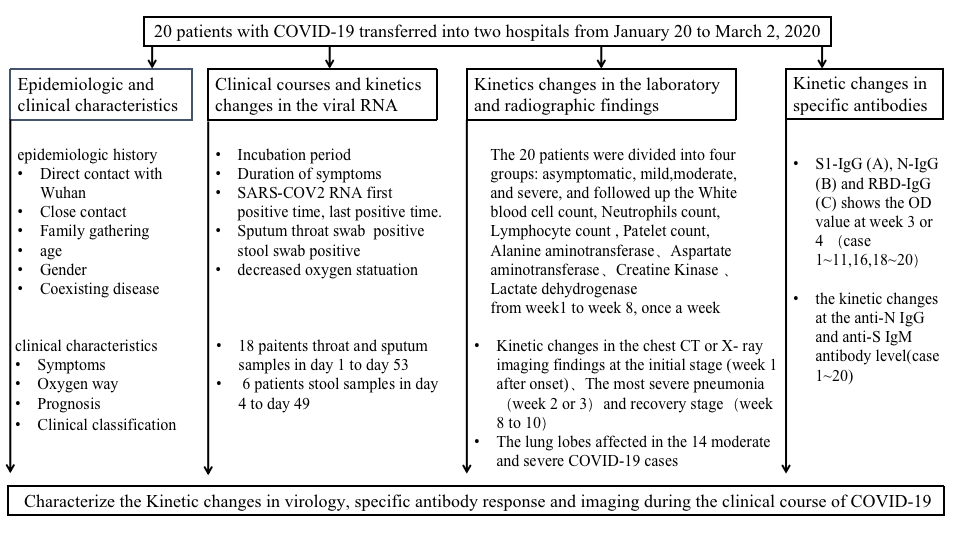

Supplement: Supplementary file 1 — Additional file 1: Supplementary Figure 1. The study design and data collection flow chart. [file 12879_2020_5549_MOESM1_ESM.docx]
